# Effects of Compound Centella on Oxidative Stress and Keap1-Nrf2-ARE Pathway Expression in Diabetic Kidney Disease Rats

**DOI:** 10.1155/2020/9817932

**Published:** 2020-05-30

**Authors:** Qin Zhu, Jiali Zeng, Jian Li, Xueming Chen, Jianxia Miao, Qinyang Jin, Hongyu Chen

**Affiliations:** ^1^Department of Nephrology, Key Laboratory of Zhejiang Province, Management of Kidney Disease, Hangzhou Hospital of Traditional Chinese Medicine, No. 453 Stadium Road, Hangzhou 310007, China; ^2^Department of Nephrology, Wenling Hospital of Traditional Chinese Medicine, No. 21 Mingyuan Road, Taizhou 317500, China; ^3^Department of Pharmacy, The Affiliated Hospital of Medical School of Ningbo University, No. 247 Renmin Road, Ningbo 315020, China; ^4^Department of Cardiology, Zhejiang Provincial People's Hospital, People's Hospital of Hangzhou Medical College, No. 158 Shangtang Road, Hangzhou 310014, China

## Abstract

The formula of Compound Centella mainly contains 3 traditional Chinese herbs: *Centella asiatica* (L.) Urb. (JiXueCao), *Astragalus Membranaceus* Fish. (HuangQi), and *Tripterygium wilfordii* Hook. f. (LeiGongTeng). Though this formula is effective for treating diabetic kidney disease (DKD) in clinic, the exact mechanism is still unclear. This study aims to investigate the effect and antioxidant mechanism of Compound Centella on DKD rats. High-performance liquid chromatography (HPLC) was applied to analyse 3 herbs in Compound Centella. Sprague-Dawley rats were divided into the normal group (NG), DKD group (DKDG), Compound Centella group (CCG), and losartan group (LG), with 8 rats in each group. On the first day of the experiment, rats in the NG were fed with ordinary –feed, while the other groups were fed with high-fat and high-sugar feed. On the 29^th^ day, except the NG, the other 3 groups received a single intraperitoneal injection of streptozocin (STZ, 35 mg/kg). Fasting blood glucose (FBG) was measured on the 1^st^ day, 32^nd^ day, 46^th^ day, 56^th^ day, 84^th^ day, and 112^th^ day. Total protein/creatinine ratio of urine was determined by the phenol red assay on the 1^st^ day and 112^th^ day. Serum creatinine (Scr) was determined by an automatic biochemical analyser on the 112^th^ day. Kidneys were collected on the 112^th^ day for analysis and evaluation. Periodic acid-Schiff (PAS) staining, hematoxylin-eosin (HE) staining, and transmission electron microscopy were used to observe kidney pathological changes. The mRNA and protein expressions of Kelch-like ECH-associated protein 1 (Keap1) and nuclear factor-erythroid 2-related factor 2 (Nrf2) in renal tissues were detected by RT-qPCR, Western blot, and immunohistochemistry. Oxidative stress was evaluated by detecting the levels of malondialdehyde (MDA) and heme oxidase-1 (HO-1). The results showed that the content of asiaticoside, astragaloside, and triptolide in the herb was 5960, 809, and 2.42 *μ*g/g and in the Compound Centella was 340, 64, and 0.1403 *μ*g/mL by HPLC. Compound Centella reduced the urine protein/creatinine ratio and improved renal pathology in the kidneys of DKD rats. In addition, the mRNA and protein expressions of Keap1 and Nrf2 in kidneys were upregulated by Compound Centella. The expressions of MDA were downregulated but HO-1 were upregulated by Compound Centella. Therefore, the protective effect of Compound Centella in the kidney of DKD rats may be related to regulating the Keap1-Nrf2-ARE pathway under oxidative stress, suggesting Compound Centella as a promising treatment against DKD.

## 1. Introduction

Diabetic kidney disease (DKD) is one of the most serious chronic complications of diabetes. In recent decades, DKD has become the main cause of the end-stage renal disease (ESRD) worldwild [1]. With the development of medicine, there are more and more ways to treat DKD, including lifestyle adjustment, glycemic control, blood pressure control, renin-angiotensin system (RAS), sodium-dependent glucose transporter-2 (SLGT-2) inhibitors, and glucagon-like peptide-1 (GLP-1) agonists [2]. Although lots of managements have been used in this disease, the clinical effect is still not satisfactory. Therefore, the discovery of new interventions that overcome these limitations to achieve delaying the development of DKD is warranted.

In China, traditional Chinese medicine (TCM) has been widely used in treating diabetes and its complications for many years. Now, TCM gets more attention from other countries and is becoming a promising new therapeutic agent for DKD [3]. One of such medicines is the Compound Centella. This formula is founded by Professor Yongjun Wang, a famous contemporary doctor of TCM. This formula has been proved to be effective for treating DKD in the clinic, but the main mechanisms are still unclear. This formula's main content is *Centella asiatica* (L.) Urb. (JiXueCao). Its extract, asiaticoside, has demonstrated several pharmacological actions such as antihyperglycemic effects in obese diabetic rats [4], restoration of the activities of kidney enzymes involved in glucose and amino acid oxidation in diabetes, and protection diabetic tissues from stress via antioxidant mechanisms in the management of DKD [5]. Oxidative stress plays a key role in the pathogenesis of DKD. The kelch-like ECH-associated protein 1 (Keap1)-nuclear factor-erythroid 2-related factor 2 (Nrf2)-antioxidant response element (ARE) pathway is the most important endogenous antioxidant stress pathway [6]; so, in the present study, the relationship between Compound Centella and the molecular mechanism of the Keap1-Nrf2-ARE pathway will be deeply explored, to provide a more experimental basis in Compound Centella for the treatment of DKD.

## 2. Materials and Methods

### 2.1. Drugs and Reagents

Losartan potassium tablets were purchased (Merck Sharp & Dohme (Australia) Pty., Ltd, South Granville, Australia, import drug registration nos. H20160398 and H20160401); streptozocin (STZ) was purchased from Sigma Chemical Co., St. Louis, USA, CAS no. 18883-66-4; Keap1 antibody was bought from Proteintech Group, Wuhan, China, no. 13161-1-ap; Nrf2 antibody was purchased from Proteintech Group, Wuhan, China, no. 16396-1-ap; heme oxidase-1 (HO-1) antibody was purchased from Proteintech Group, Wuhan, China, no. 10701-1-ap; the malondialdehyde (MDA) test box was purchased (Nanjing Jiancheng Bioengineering Institute, Nanjing, China, no. A003-1). Acetonitrile was purchased from Merck, Billerica, USA; asiaticoside reference was purchased from the National Institute for the Control of Pharmaceutical and Biological Products, Beijing, China; astragaloside reference was purchased from the National Institute for the Control of Pharmaceutical and Biological Products, Beijing, China; triptolide reference was purchased (content ≥98%, the National Institute for the Control of Pharmaceutical and Biological Products, Beijing, China).

### 2.2. Instruments

ABI 7900HT fluorescence quantitative PCR was purchased (Applied Biosystems, Foster City, USA); the Applied Biosystems LDZ 5-2 low-speed automatic balancing centrifuge was purchased (Beijing Jingli Co., Ltd., Beijing, China); the 5810R high-speed frozen centrifuge was bought (Eppendorf, Hamburg, Germany); the Bio-tek ELX800 automatic microplate reader was purchased (Bio-Tek, Biotek Winooski, USA); the Yuyou type II (301) glucose meter was purchased form Yuyue Medical Equipment & Supply Co., Ltd., Suzhou, China; ProStar 230 high-performance liquid chromatogram was purchased from Varian, Palo Alto, USA; the BX 51 optical microscope was purchased (Olympus, Tokyo, Japan); the JEM1400 transmission electron microscope was purchased (JEOL, Tokyo, Japan).

### 2.3. Animals and Experimental Design

32 male SPF Sprague-Dawley rats (4-5 weeks old; weighing 100 ± 10 g) were purchased from the Shanghai Sippr-BK Laboratory Animal Co., Ltd. These rats were bred by the animal centre laboratory of Zhejiang Chinese Medical University (Zhejiang, China) with the experimental license number of SYXK (Zhejiang) 2018-0012. And the experiment was approved by the Zhejiang Chinese Medical University Animal Ethics Committee. All rats were housed in a barrier environment (temperature, 20°C∼25°C; humidity, 50%∼65%; light, 12 h/d). The high-fat and high-sugar feed contented 10% lard, 10% sucrose, 2.0% cholesterol, 0.5% cholic acid, 5% yolk powder, and 72.5% ordinary feed formula. The high-fat and high-sugar feed's unit calorie supply is 3.95 kcal/g. The daily food for each rat was about 25∼30 g. Daily caloric intake was calculated by multiplying the mass of daily food intake in grams by the physiological fuel value of the diet in kilocalories per gram. So, the calories for each rat per day was about 98.75∼118.5 kcal. All Sprague-Dawley rats were divided into the normal group (NG), DKD group (DKDG), Compound Centella group (CCG), and losartan group (LG), with 8 rats in each group. On the first day of the experiment, 8 rats (in the NG) were fed with ordinary feed, while the other 24 rats (in the DKDG, CCG, and LG) were fed with high-fat and high-sugar feed. On the 29^th^ day of the experiment, 24 rats (in the DKDG, CCG, and LG) were given an intraperitoneal injection of STZ (dissolved in sodium citrate buffer at a concentration of 1%). The STZ dose was 35 mg/kg for a rat after fasting for 12 hours. After injection of STZ, 24 rats (in the DKDG, CCG, and LG) were continued to be fed by high-fat and high-sugar feed until the end of the experiment. On the 32^nd^ day of the experiment, all rats' fasting blood glucose (FBG) was measured via the tail vein. The diabetic rat model was successfully established when FBG ≥ 16.7 mmol/L.

### 2.4. Drug Administration

The main drugs of Compound Centella for one adult per day were as follows: *Centella asiatica* (L.) Urb. (JiXueCao) (30 g), *Astragalus membranaceus* Fish. (HuangQi) (30 g), and *Tripterygium wilfordii* Hook. f. (LeiGongTeng) (15 g). The doses of this compound for adults rely on Professor Wang's academic knowledge and clinical experience for many years. The doses for the rat can be converted from the human dose according to the body surface area formula. The body surface area was calculated based on the Meeh-Rubner equation (*A* = *k* (*W*^2/3^)/10000 (*k* = 9.1)) [7]. Therefore, the doses of herbs converted to rats were accordingly such that 0.8 g, 0.8 g, and 0.4 g. These herbs were mixed in water, decocted for 45 min, and then concentrated. The final volume of concentrated decoction was 2 mL for one rat per day. The daily treatment dose of losartan potassium tablets for per adult was 50 mg/d, which was 4.5 mg/kg/d for a rat (diluted to 2 mL with normal saline). All animals were given the drug by gavage, and the rats in the NG and DKDG were given the same dose of normal saline. All the rats were sacrificed on day 112^th^ of the experiment.

### 2.5. Specimen Collection

Urine was collected on the first day and 112^th^ day of this study. The rats were anaesthetized with pentobarbital sodium before sacrificed on the 112^th^ day. Blood was drawn from the abdominal aorta. After quick-freezing in liquid nitrogen, one side of the kidney tissues was stored in the refrigerator at −80°C, and the other side tissues were fixed in 4% paraformaldehyde and performed by paraffin embedding.

### 2.6. Chemical Analysis of Compound Centella Extracts by High-Performance Liquid Chromatography (HPLC)

#### 2.6.1. Determination of *Centella asiatica* (L.) Urb. (JiXueCao) and Asiaticoside in Compound Centella

The chromatographic conditions were as follows: column, YMC ODS C18 (4.6 mm × 250 mm, 5 *μ*m); mobile acetonitrile : water (30 : 70); atomization temperature, 40°C; gasification temperature, 90°C; flow rate, 1.0 mL/min; and nitrogen flow rate, 1.6 L/min. Preparation of reference solution was as follows: an appropriate amount of reference of asiaticoside was precisely weighed and added with methanol to make a solution containing 0.27 mg/mL. Preparation of sample solution was as follows: the herb power of *Centella asiatica* (L.) Urb. (JiXueCao) (0.5 g) was taken into a conical bottle with plug. It was added 20 mL 80% methanol and then was weighed. The solution was conducted by ultrasonic for 30 minutes. Next, it was cooled and weighed again, being made up of the lost weight with 80% methanol. In the end, the solution was shaken well, centrifuged, and filtered through a 0.45 *μ*m filter membrane. The steps of preparation of the solution of Compound Centella were as described above. Content determination was as follows: precision absorption of the reference solution of asiaticoside (5 *μ*L) and sample solution (10 *μ*L) was injected into the liquid chromatography and then was determined.

#### 2.6.2. Determination of *Astragalus membranaceus* Fish. (HuangQi) and Astragaloside in Compound Centella

The chromatographic conditions were as follows: column, YMC ODS C18 (4.6 mm × 250 mm, 5 *μ*m); mobile acetonitrile : water (35 : 65); atomization temperature, 40°C; gasification temperature, 90°C; flow rate, 1.0 mL/min; and nitrogen flow rate, 1.6 L/min. Preparation of reference solution was as follows: an appropriate amount of astragaloside was precisely weighed, and methanol was added (0.30 mg/mL). Preparation of sample solution was as follows: the herb power of *Astragalus membranaceus* Fish. (HuangQi) (4 g) was put into the extractor and steamed into the water bath. 40 mL of methanol was added. Then, this was soaked coldly overnight. Next, some methanol was added again. This solution was heated to reflux for 4 hours. Methanol was recovered and enriched to dry. The residue was added 10 mL of water and dissolved by heat. N-Butyl alcohol was used four times. Then, this solution was washed twice by ammonia and steamed. The residue was added 5 mL water to dissolve and then was passed through the D101 macroporous adsorption resin column (diameter, 1.5 cm; length, 12 cm). Then, it was eluted with 50 mL of water, 30 mL of 40% ethanol, and 80 mL of 70% ethanol in turn. The eluent was dried and dissolved in methanol and transferred to a 5 mL measuring bottle. Methanol was added to the scale of 5 mL and shaken well. The steps of preparation of the sample of Compound Centella were as described above. Content determination was as follows: precision absorption of the reference solution of asiaticoside (5 *μ*L) and sample solution (10 *μ*L) was injected into the liquid chromatography and determined.

#### 2.6.3. Determination of *Tripterygium wilfordii* Hook. f. (LeiGongTeng) and Triptolide in Compound Centella

Chromatographic conditions were as follows: column, YMC ODS C18 (4.6 mm × 250 mm, 5 *μ*m); mobile acetonitrile : water (30 : 70); detection wavelength, 220 nm; flow rate, 1.0 mL/min; and nitrogen flow rate, 1.6 L/min. Preparation of reference solution was as follows: an appropriate amount of triptolide reference was precisely weighed, and methanol was added (0.03 mg/mL). Preparation of sample solution was as follows: the herb power of *Tripterygium wilfordii* Hook. f. (LeiGongTeng) (2 g) was set in the test tube and added methanol (100.0 mL). The solution was heated to reflux for 2 hours, cooled, and then weighed, made up the lost weight with methanol, shaken, and filtered. 75 mL of the solution was taken and then dried in a water bath. It was added methanol (2.5 mL) and methylene chloride (2.5 mL) in neutral alumina (10 g, diameter 1.5 cm, and wet-packing column). The mixture of methanol and methylene chloride (1 : 3) was used for elution. The eluent was collected at 100 mL and steamed. Then, the solution was dissolved in methanol and constant volume to a 5.0 mL volumetric flask which was shaken well (through 0.45 *μ*m filter membrane filtration). The steps of preparation of the sample of Compound Centella were as described above. Content determination was as follows: precision absorption of the reference solution of asiaticoside (5 *μ*L) and sample solution (10 *μ*L) was injected into the liquid chromatography and then determined.

### 2.7. Determination of FBG, Total Protein/Creatinine Ratio of Urine, and Serum Creatinine (Scr)

FBG was measured from the tail vein on the 1^st^ day, 32^nd^ day, 42^nd^ day, 56^th^ day, 84^th^ day, and 112^th^ day. The total protein/creatinine ratio of urine was determined by the phenol red assay on the 1^st^ day and 112^th^ day. Scr was determined by an automatic biochemical analyser on the 112^th^ day.

### 2.8. The Renal Pathology Observed by Light Microscopy

The renal tissue was fixed with 10% neutral buffer formalin, dehydrated with gradient alcohol. Then, these tissues were transparentized with xylene, waxed, embedded, and sectioned. Sections (3 *μ*m thick) were stained with hematoxylin-eosin (HE) and periodic acid-Schiff (PAS) staining. The pathological changes of the kidney were observed under microscopy. The cross section yielding the maximum diameter of the glomerulus was photographed. The mesangial matrix index (MMI, in%) was calculated as the ratio of the mesangial area to the glomerular area × 100% [8].

### 2.9. The Ultrastructure of Kidney Observed by Transmission Electron Microscopy (TEM)

1 mm × 1 mm × 1 mm renal cortical tissue masses were taken, which were fixed with 2.5% glutaraldehyde fixation and 1% osmic acid fixation. After dehydration, tissues were embedded in epoxy resin. The ultrathin sections were stained with uranium acetate-lead citrate and then examined using the transmission electron microscope. The foot processes fusion rate (FPR) of the podocyte in each specimen was calculated. The curved length of the peripheral capillary basement membrane (BM) was measured on each ultramicrograph. Meanwhile, the length of all fused processes overlying the BM was also measured. Therefore, FPR was calculated using the following formula: FPR(%)=(∑L_FP_/∑L_BM_) × 100%. ∑L_FP_ is the total length of fused process; ∑L_BM_ is the total length of peripheral capillary BM [9].

### 2.10. Real-Time Quantitative PCR Method

Total RNA was extracted by the Trizol method. And the total RNA concentration was determined by a UV spectrophotometer. Then, cDNA was synthesized by reverse transcription followed by qPCR detection, synthesized by TAKARA and quality inspected. Primer sequences are shown in Table 1.

### 2.11. Western Blot Method

According to the BCA method, the total proteins were extracted and the protein contents were determined. Then, the SDS-PAGE gel was prepared. Sample buffer was added to the protein samples (50 *μ*g/lane) resolved on the immunoblots. The mixtures were denatured at 95°C for 10 minutes. The samples were added to the gel hole for electrophoresis. The protein bands were transferred to the PVDF membrane by transfer electrophoresis. Then, the sealed membranes were directly put into the working fluids of the primary antibody (Keap1 1 : 600 dilution, Nrf2 1 : 1000 dilution, and HO-1 1 : 1000 dilution). The reaction was conducted overnight at 4°C. The unbound primary antibody was washed with 1 × TBST three times. After washing, the membranes were put into the secondary antibody working fluids (1 : 10,000 dilution) for 60 minutes. The membranes were washed with 1 × TBST 3 times again. The blots were visualized using a chemiluminescence reagent kit.

### 2.12. Immunohistochemistry

Paraffin sections were taken and dewaxed to water. Antigen repair solutions were dripped on the sections and washed with PBS 3 times. Normal goat serum sealants were added to the slices and left at room temperature for 20 minutes. The first antibodies (Keap1 1 : 100 dilution and Nrf2 1 : 100 dilution) were added to the sections and washed with PBS 3 times at 4°C overnight. Secondary antibodies were added and rinsed with PBS 3 times again. Immunostaining was performed with DAB. The sections were counterstained with hematoxylin. The results were observed under the microscope, and the average optical density (AOD) values of every field were measured by Image-Pro Plus 6.0 analysis software.

### 2.13. Enzyme-Linked Immunosorbent Assay (ELISA) Method

MDA values in renal tissues were detected according to the instructions of the ELISA kit.

### 2.14. Statistical Methods

SPSS 19.0 statistical software was used. All data were presented as the mean ± standard deviation (SD). ANOVA was used to analyse the differences in the groups. *P* < 0.05 was considered statistically significant.

## 3. Results

### 3.1. The Concentration of Representative Components in Herb and Compound Centella

**Figure 1:**
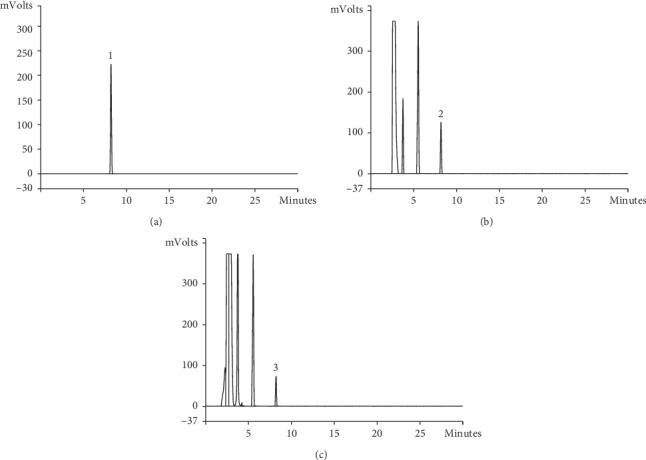
(a) Reference of asiaticoside by HPLC, (1) asiaticoside; (b) *Centella asiatica* (L.) Urb. (JiXueCao) by HPLC, (2) asiaticoside; (c) Compound Centella by HPLC, (3) asiaticoside.

**Figure 2:**
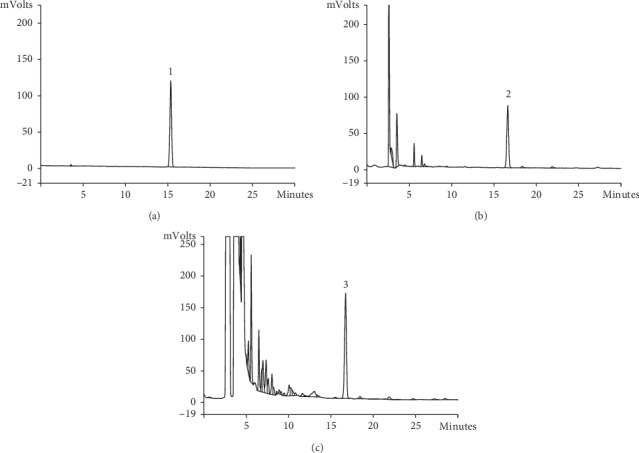
(a) Reference of astragaloside by HPLC, (1) astragaloside; (b) *Astragalus Membranaceus* Fish. (HuangQi) by HPLC, (2) astragaloside; (c) Compound Centella by HPLC, (3) astragaloside.

**Figure 3:**
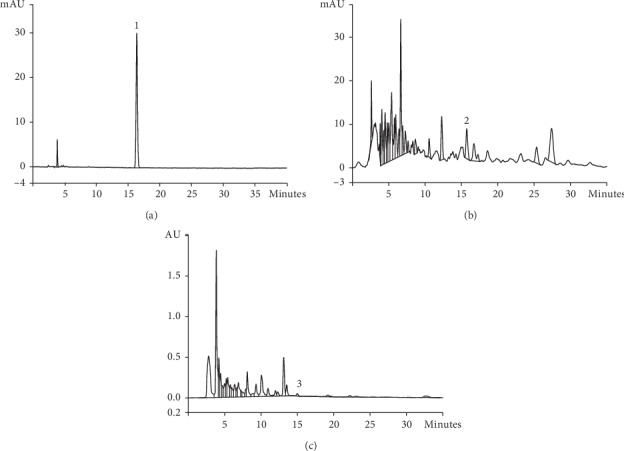
(a) Reference triptolide by HPLC, (1) triptolide; (b) *Tripterygium wilfordii* Hook. f. (LeiGongTeng) by HPLC, (2) triptolide; (c) Compound Centella by HPLC, (3) triptolide.

The representative chemical components in herb and Compound Centella were detected by HPLC. The peak retention time and concentration of asiaticoside, astragaloside, and triptolide are shown in Figures 1–3. The concentrations of representative components are shown in Table 2.

### 3.2. The Results of FBG, Protein/Creatinine Ratio of Urine, and Scr

**Figure 4:**
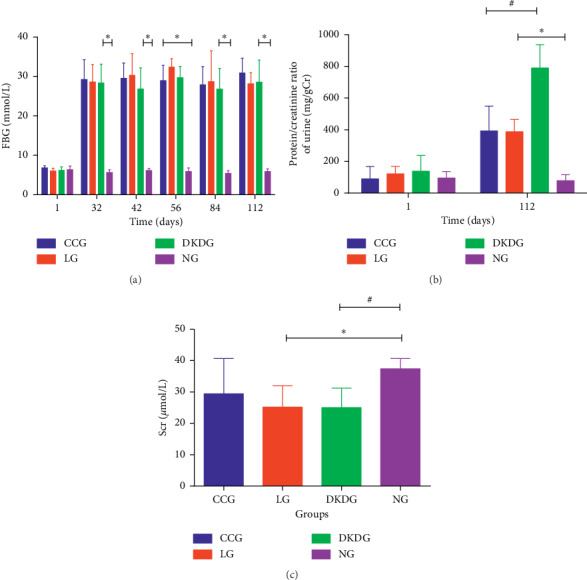
Fasting blood glucose (a), protein/creatinine ratio of urine (b), and serum creatinine (c) in 4 groups (*n* = 8 each group). Data were expressed as means ± SD. Compared with the NG, ^*∗*^*P* < 0.05. Compared with the DKDG, ^#^*P* < 0.05.

On the first day of the experiment, there was no significant difference in the FBG among four groups. On the 32^nd^ day of the experiment, compared with the NG, the blood glucose of the rats in the other groups increased significantly. And the FBG was greater than 16.7 mmol/L. However, there was no significant difference among the groups of DKDG, CCG, and LG. This trend continued until the end of the 112^th^ day of the experiment (Figure 4(a)). On the first day of the experiment, there was no significant difference in the protein/creatinine ratio of urine. On the 112^th^ day of the experiment, compared with NG, the urine protein/creatinine ratio in other groups increased significantly. Compared with the DKDG, the results in both the CCG and LG decreased significantly, but there was no significant difference between the CCG and LG (Figure 4(b)). On the 112^th^ day of the experiment, compared with the NG, the level of Scr in the DKDG was the lowest. The Scr was higher in the CCG than in the DKDG (Figure 4(c)).

### 3.3. The Results of Kidney Pathology by Light Microscopy and by TEM

**Figure 5:**
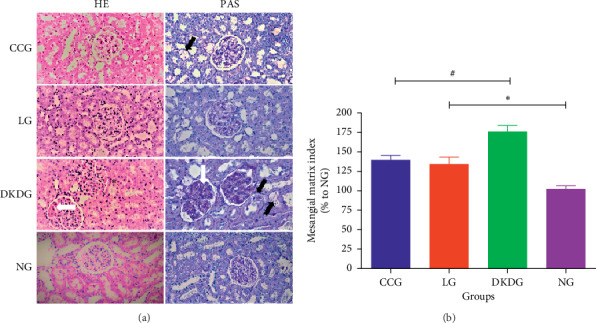
Histopathological changes of kidney staining by HE and by PAS (a) in 4 groups (*n* = 8, 400×). Black arrows indicate vacuolar degeneration of the tubules. White arrows indicate proliferated mesangial cells and stroma. Green asterisks indicate the narrow renal cavity space. The result of mesangial matrix index (% to NG) is shown in (b). Data were expressed as means ± SD. Compared with the NG, ^*∗*^*P* < 0.05. Compared with the DKDG, ^#^*P* < 0.05.

**Figure 6:**
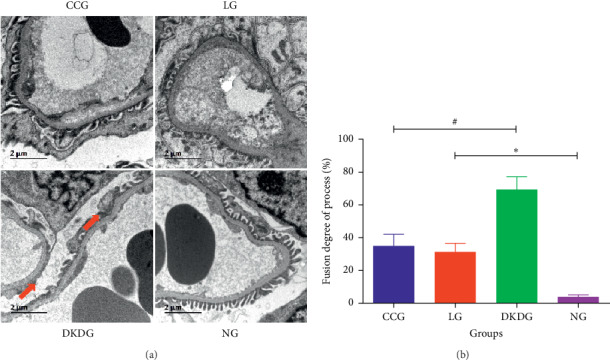
Ultrastructure changes of the kidney assayed by TEM (a) in 4 groups (*n* = 8, 10000x). Red arrows indicate foot process fusion and effacement. The results of FPR are shown in (b). Data were expressed as means ± SD. Compared with the NG, ^*∗*^*P* < 0.05. Compared with the DKDG, ^#^*P* < 0.05.

The renal tissue staining by HE and PAS showed normal appearance, with regular glomerular morphology, tight and orderly arrangement of renal tubules, and normal morphology of renal tubular epithelial cells in the NG. In the DKDG, the glomerular volume was increased and plump. The renal cavity space was narrow. The mesangial cells and stroma were proliferated. Moreover, renal tubule vacuolization was also observed in the DKDG. However, the proliferation of mesangial cells and stroma were improved in both the CCG and LG. The vacuolar degeneration of the tubules was also improved in the CCG and LG. The result showed that the mesangial matrix index reduced in the CCG and LG (Figure 5). The ultrastructure of kidney observed by TEM showed that the foot process was complete and orderly in normal rats. However, most of the foot processes were fused in the DKDG. After treatment, podocyte lesions were significantly improved in the CCG and LG. The FPR in the DKDG was significantly increased compared to the NG. After therapy, the FPR was decreased in the CCG and LG (Figure 6).

### 3.4. mRNA Contents of Keap1 and Nrf2 in Renal Tissues Tested by Real-Time Quantitative PCR

**Figure 7:**
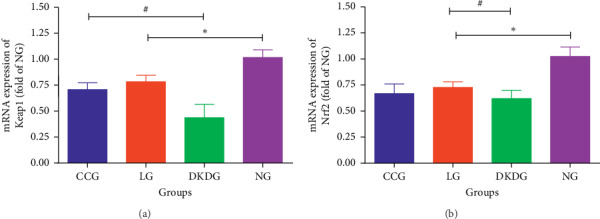
The mRNA contents of Keap1 (a) and Nrf2 (b) in renal tissues tested by real-time quantitative PCR in 4 groups (*n* = 8 each group). Data were expressed as means ± SD. Compared with the NG, ^*∗*^*P* < 0.05. Compared with the DKDG, ^#^*P* < 0.05.

mRNA expression of Keap1 was the highest in the NG but decreased significantly in the other three groups. Compared with the DKDG, Keap1 was increased in the CCG and LG. However, there was no significant difference in Keap1 between the CCG and LG (Figure 7(a)). Similarly, the mRNA expression of Nrf2 was the highest in the NG. The expression of Nrf2 mRNA in the other three groups exhibited a decreased trend (Figure 7(b)).

### 3.5. Protein Expressions of Keap1, Total Nrf2, and HO-1 in Renal Tissues Tested by Western Blot

**Figure 8:**
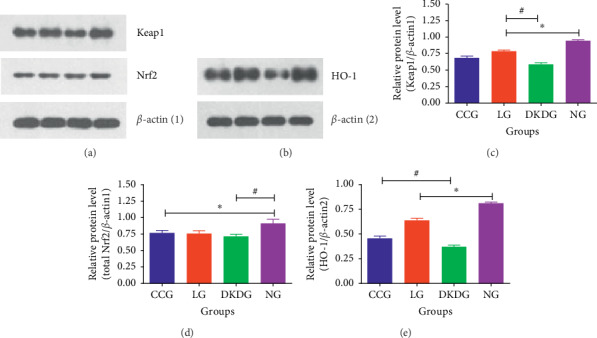
Protein expressions of Keap1 (a, c), Nrf2 (a, d), and HO-1 (b, e) in renal tissues tested by Western blot in 4 groups (*n* = 8 each group). Data were expressed as means ± SD. Compared with the NG, ^*∗*^*P* < 0.05. Compared with the DKDG, ^#^*P* < 0.05.

On the 112^th^ day of the experiment, compared with the NG, the protein expression of Keap1 in the other three groups decreased significantly. Compared with the DKDG, the protein expression of Keap1 increased in the CCG and LG. Compared with the NG, the total Nrf2 protein expression and HO-1 in the other three groups decreased. After treatment, the level of Keap1 and HO-1 had a rising trend in the CCG and LG. The level of total Nrf2 changed little after treating (Figure 8).

### 3.6. Protein Expressions of Keap1 and Nrf2 in Renal Tissues Tested by Immunohistochemistry

**Figure 9:**
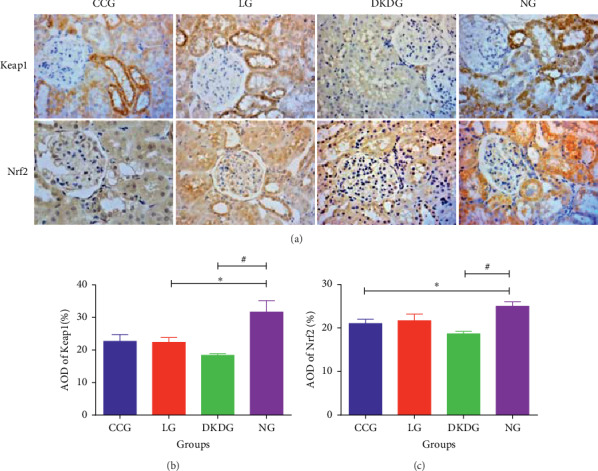
Protein expressions of Keap1 and Nrf2 (a) in renal tissues tested by immunohistochemistry in 4 groups (*n* = 8 each group) (400×). The AOD of Keap1 (b) and Nrf2 (c). Data of AOD were expressed as means ± SD. Compared with the NG, ^*∗*^*P* < 0.05. Compared with the DKDG, ^#^*P* < 0.05.

Keap1 protein-positive staining was mainly concentrated in the cytoplasm of renal tubular epithelial cells in the NG showing light yellow or brown-yellow colour, but it was not obvious in the glomerulus. Compared with the NG, it was the shallowest and least in the DKDG. The staining of Keap1 in the CCG and LG was deeper than that in the DKDG. There was no significant difference in AOD of Keap1 between the CCG and LG. Nrf2 protein-positive staining was mainly concentrated in the cytoplasm of renal tubular epithelial cells and glomerular cells showing light yellow or brown-yellow colour in the NG. Compared with the NG, the other three groups showed shallower positive staining and the site of Nrf2 expression changed from cytoplasm mainly to not only cytoplasm but also nucleus. It was the least and shallowest in the DKDG compared with the DKDG. The staining of the Nrf2 protein was slightly deeper in the CCG and LG. There was no statistical difference in the positive staining of AOD between the CCG and LG (Figure 9).

### 3.7. MDA in Renal Tissues Determined by ELISA

**Figure 10:**
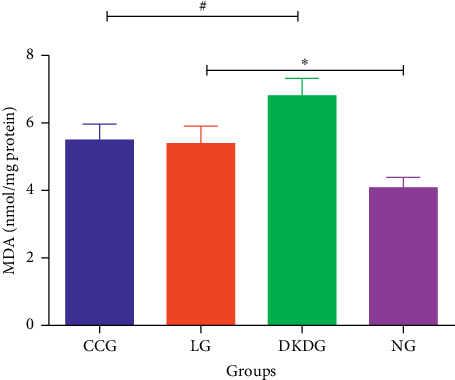
MDA in renal tissues determined by ELISA in 4 groups (*n* = 8). Data were expressed as means ± SD. Compared with the NG, ^*∗*^*P* < 0.05. Compared with the DKDG, ^#^*P* < 0.05.

The MDA in renal tissues was the lowest in the NG, and the other three groups had different degrees of increase. Compared with the CKDG, MDA decreased in the CCG and LG, but there was no significant difference between the CCG and LG (Figure 10).

## 4. Discussion

In the past decade, the incidence of dialysis has stabilized both in the general population and in diabetics, but in whom it remains far higher by comparison [7]. Therefore, it is important to clarify the pathogenesis of DKD as early as possible and to find effective methods to delay the progress of DKD. At present, the pathogenesis of DKD mainly focuses on metabolism, hemodynamics, inflammatory response, immune system, protein kinase, and oxidative stress [8–11]. In this study, a DKD rat model was established by a single intraperitoneal injection of STZ combined with a sustained high-fat and high-sugar diet. The results showed that blood glucose sustained at the level of the diabetes mellitus after modelling, indicating that this method was successful. This study found an interesting result that the protein/creatinine ratio of urine was the highest but the level of Scr was the lowest in the DKDG. How would we explain this result under this model? It is well known that the pathological process of DKD includes glomerular hypertrophy, thickening of the basement membrane and increased mesangial matrix, typical nodular glomerular sclerosis, and finally extensive glomerular sclerosis [10]. In the early stage of DKD, the glomerular filtration rate (GFR) is usually higher than normal, so the Scr is usually lower than normal. In stages 4-5, the GFR continues to decrease but the Scr continues to increase. In our study, the pathology in DKDG showed the mesangial matrix and mesangial cell proliferation without classic Kimmelstiel-Wilson nodules or obvious glomerular sclerosis or tubulointerstitial fibrosis, which phenomenon is mainly on 2-3 stages according to the Mogensen stage [11]. This outcome supports the kidney disease of diabetic rats in our study were in the early stage of DKD. So, the phenomenon was reasonable that the level of Scr was lowest in the DKDG.

It is now believed that oxidative stress is a common factor in many pathogeneses of DKD [12]. Living in an oxygenated environment has required the evolution of effective cellular strategies to detect and detoxify metabolites of molecular oxygen known as reactive oxygen species (ROS). When the body is exposed to various harmful stimulations, the body will produce too many ROS to remove it, resulting in an imbalance between the oxidative system and the antioxidant system. Then, the body tissues will be damaged. This process is called oxidative stress [13]. The Keap1-Nrf2-ARE signalling pathway is considered to be the most important pathway in the antioxidant mechanism. Nrf2 is a redox transcription factor and a receptor for oxidative stress, playing an important role in regulating the basal and inducible expression of numerous detoxifying and antioxidant genes. Nrf2 is expressed in various tissues such as the kidney, liver, spleen, and heart. In the physiological state, Nrf2 is tethered by a cytosolic protein Keap1, which targeted for ubiquitin-dependent proteasomal degradation. Keap1 is the main cytosolic inhibitor of Nrf2 and possesses several Cys residues. Modification of these residues by ROS and electrophilic agents leads to spatial alterations in the Nrf2-Keap1 complex. Upon exposure to oxidative stress, Nrf2 is dissociated from Keap-1 and rapidly translocated into the nucleus and then binds with ARE. It results in the transcriptional activation of the cell defence system [14, 15]. This defence system mainly includes but not limited to the following: (1) as a scavenger of free radicals, the SOD content indirectly reflects the body's ability to remove oxygen free radicals; (2) HO-1 is an important anti-inflammatory enzyme that protects the body from both oxidative stress and toxic substances [16]. These free radical scavenging enzymes form a powerful antioxidant defence system. In contrast, NADPH oxidase 4 (NOX4) is one of the NADPH oxidase subunits, the primary sources of free radicals in the cell [17]. Prior studies have noted that the NADPH oxidase including NOX4 could cause renal tissue damage in diabetes [18]. MDA is the product of lipid peroxidation. Its content reflects the level of lipid peroxidation and the severity of the free radical attack on cells, which is considered an independent risk factor of DKD [19]. Our results are in accord with previous studies that after modelling, the expression level of SOD and HO-1 was decreased but MDA was increased in diabetic rats compared to normal ones. It suggests that oxidative stress injury occurred and the antioxidant defence system is inhibited in DKD.

Currently, although some scholars have published articles on Keap1 or Nrf2, the research results are still controversial. It is hard to explain those results, especially in the presence of complex diseases such as diabetes. In an *in vivo* study, one has documented the levels of total Nrf2 and its downstream target HO-1 in renal tissues detected by ELISA were reduced in STZ-induced diabetic mice [20]. Another study showed a similar result that the total expression of Nrf2 had the descending trend in ischemia/reperfusion-induced myocardial injury diabetic mice [21]. Once the Nrf2 gene was knocked out, the mice produced more ROS in the kidney and severe DNA oxidative damage than normal mice [22]. In addition, the total Nrf2 levels displayed invisible change when glomerular mesangial cells (GMCs) were challenged with advanced glycation end products (AGEs) [23]. After treatment with some antioxidants, the total protein expression of Nrf2 and its downstream targets such as SOD and HO-1 were significantly higher in diabetic animals. However, the transfection of Nrf2 siRNA abolished the expression of Nrf2-related proteins and the cytoprotective effect of antioxidants [21]. Interestingly, other researchers obtained an opposite outcome when they tested the nuclear expression of Nrf2 in diabetic models. One study found that the nuclear content of Nrf2 increased with a paramount upregulation in GMCs stimulated by AGEs [23]. A slight increase in the nuclear Nrf2 level was observed in the kidney of diabetic rats. Antioxidants could sharply increase nuclear Nrf2 levels [8]. As for the primary negative regular of Nrf2, the Keap1 expression exhibited a declining trend when GMCs were challenged with AGEs [23]. The *in vivo* study showed a similar result that the diabetic rats showed a declining trend of the Keap1 protein level in the kidney [8]. The results of this study showed that the levels of Keap1 and total Nrf2 both decreased in diabetic rats compared to normal. Although we did not analyse the nuclear Nrf2 by Western blot, we observed the location and positive staining of Nrf2 by immunohistochemistry. The AOD of total Nrf2 was descended while the positive staining location of Nrf2 changed from cytoplasm mainly to not only cytoplasm but also nucleus in diabetic modelling. From literatures and our results, we speculate that the slightly increased nuclear Nrf2 and decreased Keap1 could be considered as an adaptive response to oxidative stress. However, it is believed that diabetes is a complex and progressive disease, existing chronic inflammation and oxidative stress. When the oxidative stress associated with diseases exceeds the ability of the body's self-repair, the total Nrf2 may decline. As we know, under oxidative stress, Nrf2 will be dissociated from Keap1 and translocated into the nucleus and then activate downstream target genes. Therefore, the expression of nuclear Nrf2 may inversely increase under the station of oxidative stress.

After treatment with Compound Centella, the urine protein/creatinine ratio was lower in diabetic rats. Furthermore, the DKD renal pathology was improved. These suggest that Compound Centella is effective for DKD. We also found the expression of Keap1, as well as total Nrf2, increased after treating with Compound Centella. The expression of MDA was downregulated and HO-1 was upregulated in the CCG. And in the group of positive control drug, losartan had a similar trend. It is suggested that Compound Centella may ameliorate DKD by activating the Keap1-Nrf2-ARE pathway and regulate oxidative stress.

This study has a few limitations. First, the nuclear content of Nrf2 has not yet been elucidated. Second, which one is the main role in Compound Centella working as an antioxidant is not confirmed. Therefore, the single component in Compound Centella and the nuclear Nrf2 protein must be explored in subsequent studies.

## 5. Conclusion

In conclusion, Compound Centella can reduce the urine protein/creatinine ratio and improve renal pathology in the kidneys of DKD rats. Also, the mRNA and protein expression of Keap1 and total Nrf2 in kidneys were upregulated by Compound Centella. The expression of MDA was downregulated but HO-1 was upregulated in the kidneys of DKD rats. Therefore, the protective effect of Compound Centella in the kidney of DKD rats may be caused by regulating the Keap1-Nrf2-ARE pathway in the oxidative stress pathway, suggesting a further investigation of Compound Centella as a promising treatment against DKD.

## Figures and Tables

**Table 1:** Primer sequences used for the quantitative real-time polymerase chain reaction.

Gene	Forward primer (5′-3′)	Reverse primer (5′-3′)
Keap1	tgctcaaccgcttgctgtatgc	tcatccgccactcattcctctcc
Nrf2	gccttcctctgctgccattagtc	tcattgaactccaccgtgccttc
GAPDH	acagcaacagggtggtggac	tttgagggtgcagcgaactt

**Table 2:** The concentration of representative components in the herb and in the Compound Centella.

Standard substance	Concentration in the herb (*μ*g/g)	Concentration in the Compound Centella (*μ*g/mL)
Asiaticoside	5960	340
Astragaloside	809	64
Triptolide	2.42	0.1403

## Data Availability

The data used to support the findings of this study are available from the corresponding author upon request.

## Abbreviations

AGEs: Advanced glycation end products
ARE: Antioxidant response element
AOD: Average optical density
BM: Basement membrane
DAB: 3,3-Diaminobenzidine
DKD: Diabetic kidney disease
ESRD: End-stage renal disease
FBG: Fasting blood glucose
FPR: Foot processes fusion rate
GFR: Glomerular filtration rate
GLP-1: Glucagon-like peptide-1
GMCs: Glomerular mesangial cells
HO-1: Heme oxidase-1
HPLC: High-performance liquid chromatography
Keap1: Kelch-like ECH-associated protein 1
MDA: Malondialdehyde
MMI: Mesangial matrix index
NOX4: NADPH oxidase 4
Nrf2: Nuclear factor-erythroid 2-related factor 2
RAS: Renin-angiotensin system
ROS: Reactive oxygen species
SGLT-2: Sodium-dependent glucose transporter-2
SOD: Superoxide dismutase
STZ: Streptozocin
TEM: Transmission electron microscopy
TCM: Traditional Chinese medicine.
